# Comparison of GPT-4 omni and physicians for accurate evaluation of traumatic extremity x-rays: A feasibility study

**DOI:** 10.1097/MD.0000000000048176

**Published:** 2026-03-27

**Authors:** İlter Ağaçkiran, Merve Ağaçkiran

**Affiliations:** a Department of Emergency Medicine, School of Medicine, Hitit University, Corum, Turkey; b Department of Emergency Medicine, Corum Erol Olcok Training and Research Hospital, Corum, Turkey.

**Keywords:** fracture, GPT-4 omni, trauma, x-ray

## Abstract

GPT-4 omni (GPT-4o) is the latest version of Chat-GPT, developed by OpenAI, which provides image interpretation capabilities. In trauma patients, x-rays are the simplest imaging method, and research is ongoing on their interpretation using artificial intelligence. No published studies have evaluated the accuracy of GPT-4o in x-ray interpretation; hence, the aim of this study was to determine the diagnostic accuracy of GPT-4o in x-ray interpretation and compare its performance with that of emergency medicine specialists, residents, and orthopedists. Ten cases were randomly selected from the Northwestern University Feinberg School of Medicine Orthopedic Teaching page and presented to the groups, and the results were recorded. In the research, emergency medicine assistants, emergency medicine specialists, orthopedic specialists and GPT-4o were asked about these ten cases separately in Turkey between June 1, 2024 and July 15, 2024. Ten individuals from each group and the answers to questions asked on GPT-4o different days were included in the study. There was no significant difference between GPT-4o and emergency medicine residents, whereas a significant difference was observed between these 2 groups and between emergency medicine specialists and orthopedists (*P* = .017). Orthopedists had the highest accuracy rate (86%), whereas emergency medicine specialists, emergency medicine residents, and GPT-4o had accuracy rates of 84%, 76%, and 74%, respectively. Overall abilities of ChatGPT-4o as a general diagnostic tool is less successful in x-ray interpretation than physicians’ interpretation. The accuracy increases with the removal of dislocations from the data pool. These results suggest that GPT-4o can assist physicians only in cases of suspected fractures.

## 1. Introduction

The term artificial intelligence (AI) was coined by John McCarthy and is currently used as a general definition for machines (computers) that mimic or simulate human intelligence, encompassing their capabilities and what they can achieve.^[1,2]^ Recently, studies demonstrating the development of AI’s ability to make complex interpretations at the level of healthcare professionals have been rapidly increasing.^[3-8]^ However, it has been predicted that AI could replace healthcare professionals, particularly radiologists, causing concerns among healthcare teams.^[9]^ With the development of AI, it may be possible to interpret radiographs in small hospitals without radiologists or orthopedists.^[8]^

The Chat-GPT is a well-known AI model with ~100 million active users.^[10]^ Using GPT-4 omni (GPT-4o), an update of Chat-GPT, the ability to upload and interpret medical images was introduced.^[11]^ This has made it easier for individuals, particularly healthcare professionals, to access AI. GPT-4o is the latest version of OpenAI, and there are no studies on its ability to diagnose pathologies on trauma radiographs, and its level of success in this task is not yet known.

This study aimed to evaluate the diagnostic accuracy of GPT-4o in interpreting x-ray images accompanied by brief clinical information and to compare the diagnostic accuracies of 4 groups: emergency medicine specialists, emergency medicine residents, orthopedists, and GPT-4o.

## 2. Materials and methods

The study was conducted between June 1, 2024, and July 15, 2024, in Turkey, and included emergency medicine residents, emergency medicine specialists, orthopedic specialists, and GPT-4o. This study did not include patient data; hence, ethics committee approval was not required. Ten individuals each from emergency medicine residents, specialists, and orthopedic specialists were included. Additionally, GPT-4o was used to evaluate each case 10 times. Participants were recruited voluntarily from the departments of emergency medicine and orthopedics. Each group consisted of 10 physicians. Specialists had completed residency training, while residents were in different stages of their training. Detailed data on years of clinical experience or prior exposure to the specific teaching cases were not collected, which represents a limitation of the study.

Ten cases were randomly selected from the Northwestern University Feinberg School of Medicine Orthopedic Teaching page.^[12]^ The cases included 3 radiographs of the wrist (including 1 pediatric case), 2 of the pelvic, 2 of the knee, 2 of the ankle, and 1 of the elbow. The cases were presented with brief anamnesis, as described on the website. The initial responses were collected “What is the diagnosis?,” open-ended answers. The second question regarding how patient management in the emergency department should be (to determine if patients need emergency surgery) was presented in the multiple-choice test format. (The questions of the study are available in the Supplementary File 1. Please see, Supplemental Digital Content, https://links.lww.com/MD/R584) The participating physicians were asked to evaluate the cases. Written informed consent was obtained from all the participants. Cases were sent to the participants via Google Docs, and 30 minutes were given to evaluate each case. Thereafter, the responses were recorded. The results were compared with the correct answers on the Northwestern University Feinberg School of Medicine Orthopedic Teaching page (Table 1).

**Table 1:** Examining the number of correct numbers within and between groups.

	Area of specialization				Test stat.	*P* *
	Emergency medicine specialist (n = 10)	Emergency medicine resident (n = 10)	Orthopedics and traumatology specialist (n = 10)	Chat-GPT (n = 10)
Correct answer- x-ray	9 (7–10)‡	8 (6–9)‡	9 (6–10)‡	8 (6–8)‡	10.144	**.017**
Correct answer – emergency surgery	8.5 (7–10)‡	8 (7–9)‡	10 (9–10)‡	9 (8–10)‡	18.323	**<.001**
Test stat.	13.000	31.500	25.000	55.000		
*P* †	.589	.248	.054	**.004**		

Bold values indicate statistically significant differences between groups (*P* < .05).

* Kruskal–Wallis *H* test.

† Wilcoxon test.

‡ Groups sharing the same superscript letter are not significantly different from each other based on post hoc Dunn test results with Bonferroni correction. Median (min–max).

The cases were presented in the format on the web page, that is, radiographs and the case history were given together. Before the cases were asked, GPT-4o was asked to act like a physician working in an emergency department. Ten different chats were created on different days, and all of them were asked to be emergency department physicians. In each chat, ten cases were asked in a different order. GPT-4o resolved the cases and provided explanations regarding the necessity of emergency surgical operations. The full prompt used for GPT-4o is provided in the supplementary materials for reproducibility. GPT-4o was asked to evaluate each case 10 times between June 3, 2024, and June 15, 2024, and the results were recorded. Participants were not informed about the specific aim of comparing their performance with GPT-4o, in order to minimize bias. GPT-4o was prompted 10 separate times for each case, while each physician evaluated each case once. This asymmetry in evaluation frequency may have introduced bias in favor of the AI model and should be considered when interpreting the results.

The initial responses were collected as short open-ended answers, and the second question was asked in a multiple-choice test format, namely how patient management should be in the emergency department. While physicians were grouped according to their specialization and level of training, GPT-4o was considered a separate group, and the results were compared. The reference standard for correct answers was the teaching notes provided on the Northwestern University Feinberg School of Medicine Orthopedic Teaching page. No independent consensus review by radiologists or orthopedic surgeons was performed.

### 2.1. Statistical analyses

Data analyses were performed using IBM SPSS version 23 (SPSS Inc., Armonk) software. The normality of the variables was analyzed using the Shapiro–Wilk test. To compare non-normally distributed correct answer counts across specialty areas, the Kruskal–Wallis *H* test was used, followed by post hoc pairwise comparisons using the Dunn test with Bonferroni correction. The Wilcoxon test was used to compare non-normally distributed correct answer counts between the groups. The relationship between non-normally distributed correct answer counts within and across groups was examined using the Spearman’s rho correlation coefficient. The results are presented as median, minimum, and maximum (median [min–max]). The significance level was set at *P* < .05.

## 3. Results

GPT-4o answered an average of 7.4 of the 10 questions correctly in the cases, resulting in an accuracy rate of 74%. Emergency medicine specialists answered an average of 8.4 of the 10 questions correctly, emergency medicine residents answered 7.6, and orthopedists answered 8.6, resulting in accuracy rates of 84%, 76%, and 86%, respectively (Supplemental digital content, Supplemental Digital Content, https://links.lww.com/MD/R584). Significant differences were observed in the number of correct answers given to cases across the specialty areas (*P* = .017). This difference was observed between orthopedics and traumatology specialists and the GPT-4o. The median number of correct answers for orthopedics and traumatology specialists was 9, whereas it was 8 for GPT-4o. Based on these results, the number of correct answers by GPT-4o was lower than those of orthopedics and traumatology specialists.

A significant difference was observed between the number of correct answers given to the question regarding the necessity of emergency surgery across specialty areas (*P* < .001), and this difference was observed between orthopedics and traumatology specialists and between GPT-4o and emergency medicine residents. The median number of correct answers was 10 for orthopedics and traumatology specialists, 9 for GPT-4o, and 8 for emergency medicine residents. In line with these results, the number of correct answers by orthopedic and traumatology specialists and GPT-4o was higher than that of emergency medicine residents.

A significant difference was found between the number of correct answers given by GPT-4o to the cases and the responses to questions about the necessity of emergency surgery (*P* = .004). The median number of correct answers given by GPT-4o to the cases was 8, whereas the median number of correct answers given to questions about the necessity of emergency surgery was 9. In line with these results, the number of correct answers given by GPT-4o to questions regarding the necessity of emergency surgery was higher. There was no significant difference between the number of correct answers given to the cases and the number of correct answers given to the questions about the necessity of emergency surgery by emergency medicine specialists, emergency medicine residents, and orthopedics and traumatology specialists (*P* > .05; Table 1).

There was no significant correlation between the number of correct answers given to the cases and the number of correct answers given to the questions regarding the necessity of emergency surgery within the specialty areas (*P* > .05). A moderate positive correlation was found between the number of correct answers given to the cases and the number of correct answers given to the questions on the necessity of emergency surgery (*r* = 0.401, *P* = .010) regardless of specialty (Table 2).

**Table 2:** Examining the relationship between the number of correct answers given to cases within and without groups and the number of correct answers given to emergency surgery cases.

	*r*	*P*	N
Emergency medicine specialist	0.602	.065	10
Emergency medicine resident	0.457	.184	10
Orthopedics and traumatology specialist	−0.282	.429	10
Chat-GPT	0.456	.185	10
Total	0.401	**.010**	40

Bold values indicate statistically significant differences between groups (*P* < .05).

*r*: Spearman’s ρ correlation coefficient.

There was no statistically significant relationship between the number of correct answers given to cases A and B within and across groups (*P* > .05; Table 3).

**Table 3:** Examining the relationship between the number of correct answers given to cases A and B within and across groups.

	*r*	*P*	N
Emergency medicine specialist	0.514	.129	10
Emergency medicine resident	0.361	.306	10
Orthopedics and traumatology specialist	0.091	.802	10
Chat-GPT	0.199	0.581	10
Total	0.277	0.084	40

*r*: Spearman’s ρ correlation coefficient.

## 4. Discussion

In this study, the accuracy performance of GPT-4o, the latest version of Chat-GPT, was evaluated in interpreting x-ray images in traumatic cases and compared with than of emergency medicine specialists, emergency medicine residents, and orthopedics and traumatology specialists. To our knowledge, there are not enough studies on this subject in the literature.

In this study, GPT-4o performed equally well as emergency medicine residents in interpreting x-rays of patients with trauma; however, it gave fewer correct answers compared to orthopedists and emergency medicine specialists. Previous versions of Chat-GPT have been successfully used in many medical examinations.^[13-17]^ Chung et al demonstrated a 95% accuracy rate in AI interpretation of x-ray images for proximal humerus fractures, whereas Cheng et al reported a 91% accuracy rate for hip fractures.^[18,19]^ In this study, we found a 74% accuracy rate, with an average of 7.4 correct answers for 10 questions. This difference may stem from the use of machine learning, which has been specifically applied to x-ray imaging in previous studies. The GPT-4o is a general AI model that is not specialized. When examining the accuracy rates among the groups, orthopedics and traumatology specialists achieved the highest accuracy rate of 86%, followed by emergency medicine specialists and emergency medicine residents at 84% and 76%, respectively. Among the 4 groups, the GPT-4o had the lowest accuracy rate.

GPT-4o correctly identified the ankle, carpal, and femoral shaft fractures. Although it accurately interpreted the images, it provided incorrect treatment-planning responses. This indicates that, although GPT-4o could establish an accurate diagnosis when interpreting the x-ray, it could not determine whether there was displacement at certain instances. Providing detailed physical examination information can yield more accurate results. One case in which GPT-4o was less successful than the other groups was hip dislocation. In this case, GPT-4o gave correct answers 7 of the ten times. Upon reviewing the incorrect answers, GPT-4o was found to be confused with a femoral neck fracture.

An interesting finding of this study was that GPT-4o performed better in determining the need for emergency surgery (median = 9) than in establishing the primary diagnosis (median = 8). This suggests that although the model may lack full diagnostic precision, it is able to recognize high-acuity patterns that warrant urgent intervention. One possible explanation is that GPT-4o integrates the clinical context cues from the case descriptions and tends to adopt a risk-averse approach when faced with potential high-risk scenarios. This feature may represent a relative strength of the model, indicating a potential role in triage or as a supplementary tool to flag cases requiring urgent surgical evaluation. However, these findings must be interpreted cautiously given the limited sample size.

In this study, the most frequently misinterpreted case was an image showing a scaphoid fracture accompanied by a perilunate dislocation. This was the case with the lowest accuracy rate among all the groups. Despite being asked 10 times, GPT-4o failed to provide the correct answer in any instance, demonstrating the poorest performance among the groups. Upon review of the literature, the incidence of perilunate dislocation is unknown because it is usually overlooked. It is rare compared to other carpal fractures; however, it is the most common dislocation of the wrist^[20,21]^ For proximal fibula fractures, all emergency medicine specialists answered correctly, whereas most orthopedics and traumatology specialists and emergency medicine residents answered correctly. However, GPT-4o missed the fracture line every time such a case was considered and interpreted as a bone contusion. Overall, GPT-4o struggled significantly in recognizing dislocations. When dislocations were excluded, the accuracy of GPT-4o increased to 83.75%. In particular, GPT-4o misinterpreted the perilunate dislocation case. We conclude that GPT-4o requires further improvement in this regard. Given the small number of cases, the statistical analyses should be interpreted with caution. Non-parametric tests applied to such a limited dataset yield only exploratory insights, and multiple comparisons increase the risk of type I error. Reported *P*-values should therefore not be over-interpreted. Presenting confidence intervals would provide additional context, but their width would likely remain large due to the limited sample size.

The most significant limitation of our study was the absence or limited availability of physical examination findings. In patients with trauma, the area requiring the most careful x-ray examination is usually identified through physical examination. This may have made it difficult for physicians to diagnose the condition. However, because the information in the cases was uniformly presented to the participants, we believe that it did not affect the outcome. The statistical analyses are constrained by the small sample size, which limits the robustness of non-parametric tests and inflates the risk of spurious significance. Another important limitation is the absence of a formal power calculation. Because the study included only 10 radiographs evaluated by 10 participants in each group, the statistical power was inherently low. Therefore, the results should be considered preliminary and hypothesis-generating rather than definitive. In addition, the study was based on only 10 radiographs, all selected from a single online teaching repository. This very limited sample may not reflect the complexity and heterogeneity of real-world trauma radiographs, which often include lower-quality images, overlapping injuries, or atypical presentations. With such a small number of cases, the findings are highly susceptible to distortion by outlier cases and should not be generalized. We suggest interpreting this study as a pilot feasibility study, providing early insights that require confirmation in larger datasets. Another limitation is that the reference standard relied on the teaching file notes rather than an independent consensus of radiology or orthopedic experts. This may weaken the reliability of the ground truth used for comparison.

## 5. Conclusions

GPT-4o’s performance in interpreting x-rays of trauma patients was comparable to that of emergency medicine residents, but less accurate than orthopedics and traumatology specialists and emergency medicine specialists. GPT-4o showed similar performance to the other groups in identifying fractures; however, it demonstrated poorer performance compared to the other groups in identifying dislocations.

## Author contributions

**Conceptualization:** İlter Ağaçkiran, Merve Ağaçkiran.

**Data curation:** İlter Ağaçkiran, Merve Ağaçkiran.

**Formal analysis:** İlter Ağaçkiran.

**Funding acquisition:** İlter Ağaçkiran.

**Investigation:** İlter Ağaçkiran.

**Methodology:** İlter Ağaçkiran, Merve Ağaçkiran.

**Project administration:** İlter Ağaçkiran, Merve Ağaçkiran.

**Resources:** İlter Ağaçkiran, Merve Ağaçkiran.

**Software:** İlter Ağaçkiran.

**Supervision:** İlter Ağaçkiran, Merve Ağaçkiran.

**Validation:** İlter Ağaçkiran.

**Visualization:** İlter Ağaçkiran.

**Writing – original draft:** İlter Ağaçkiran, Merve Ağaçkiran.

**Writing – review & editing:** İlter Ağaçkiran, Merve Ağaçkiran.

## Supplementary Material


