# Supplementary material for: Comparison of GPT-4 omni and physicians for accurate evaluation of traumatic extremity x-rays: A feasibility study
Source: Medicine (Baltimore). 2026 Mar 27;105(13):e48176. doi: 10.1097/MD.0000000000048176 (PMC13034909; doi:10.1097/MD.0000000000048176)
Supplement: Supplementary file 1 [file medi-105-e48176-s001.docx]

**Number of correct answers by cases and groups**

| **Cases** | **EMS** | **EMR** | **OTS** | **GPT** |
| --- | --- | --- | --- | --- |
| **Case1a:** A 35-year-old female complains of swelling and pain in her lateral right ankle after twisting it while walking on an uneven sidewalk at night. What is the specific diagnosis?  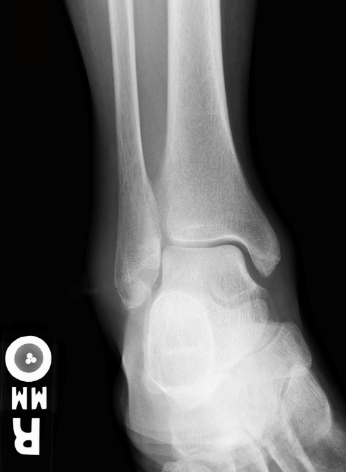  **Answer: Weber A (Lateral Malleolus/Distal Fibula) Fracture** | 7/10 | 9/10 | 8/10 | 10/10 |
| **Case 1b:** How should the emergency management of the above case be?  a. Emergency surgery should be planned.  **b. The extremity should be immobilized and the patient should be referred to an orthopedist in 1–2 weeks.** | 10/10 | 9/10 | 10/10 | 8/10 |
| **Case 2a:** A 27-year-old male presents with right ankle pain after a fall. What is the diagnosis and management?  **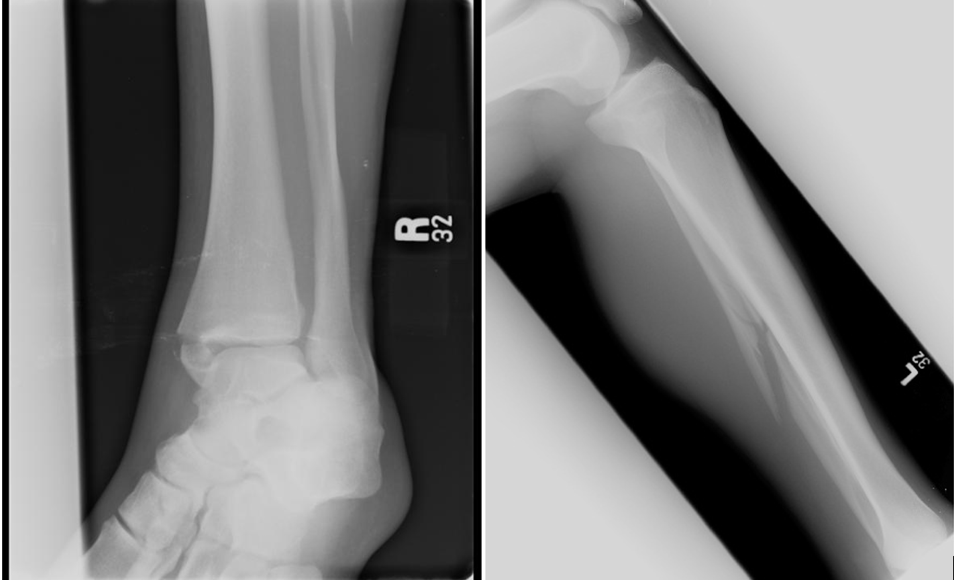**  **Answer: Maisonneuve fracture (Displaced fracture of the medial malleolus with spiral fracture of the mid-fibular shaft)** | 7/10 | 3/10 | 7/10 | 10/10 |
| **Case 2b:** How should the emergency management of the above case be?  **a. Emergency surgery should be planned.**  b. The extremity should be immobilized and the patient should be referred to an orthopedist in 1–2 weeks. | 7/10 | 6/10 | 10/10 | 10/10 |
| **Case 3a:** A 26-year-old male presents complaining of right wrist pain after falling on an outstretched hand. What is the diagnosis?  **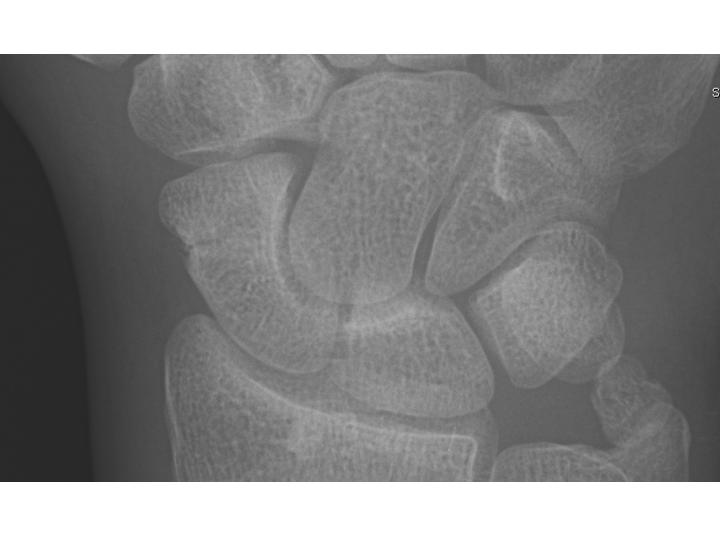**  **Answer:** **Scaphoid Fracture** | 9/10 | 8/10 | 9/10 | 10/10 |
| **Case 3b:** How should the emergency management of the above case be?  a. Emergency surgery should be planned.  **b. The extremity should be immobilized and the patient should be referred to an orthopedist in 1–2 weeks.** | 7/10 | 6/10 | 9/10 | 10/10 |
| **Case 4a:** This patient had a fall on an outstretched hand and pain over the dorsal wrist. What is the diagnosis?  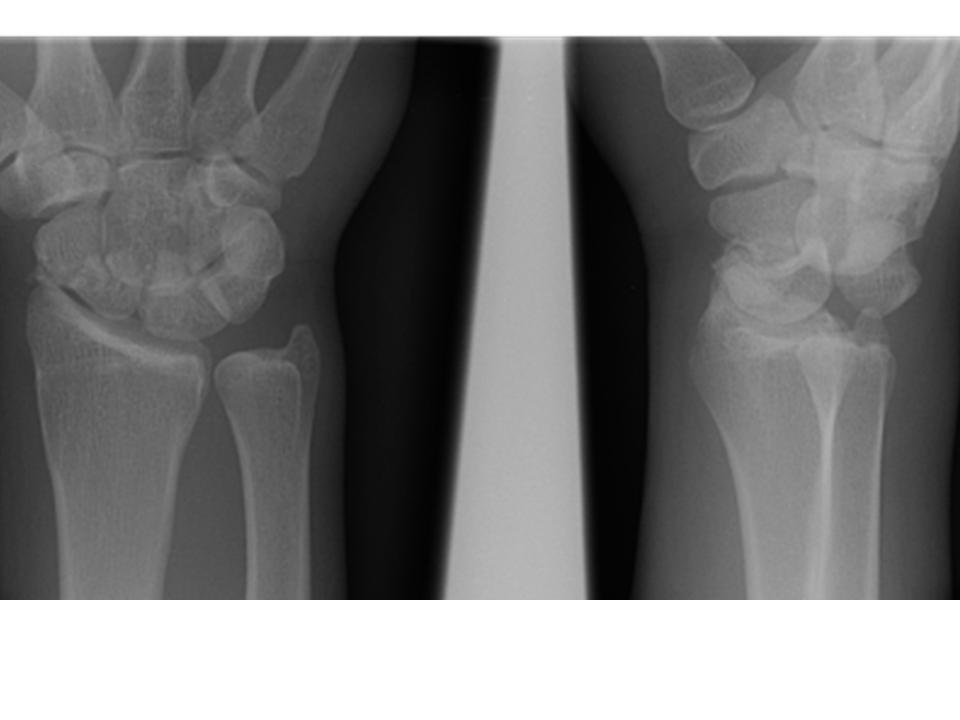  **Answer:** **Perilunate Dislocation with associated Scaphoid fracture** | 3/10 | 3/10 | 7/10 | 0/10 |
| **Case 4b:** How should the emergency management of the above case be?  **a. Immediate reduction is required.**  b. The extremity should be immobilized and the patient should be referred to an orthopedist in 1–2 weeks. | 6/10 | 6/10 | 10/10 | 5/10 |
| **Case 5a:** This patient had an MVC and has severe right hip pain. What is the diagnosis?  **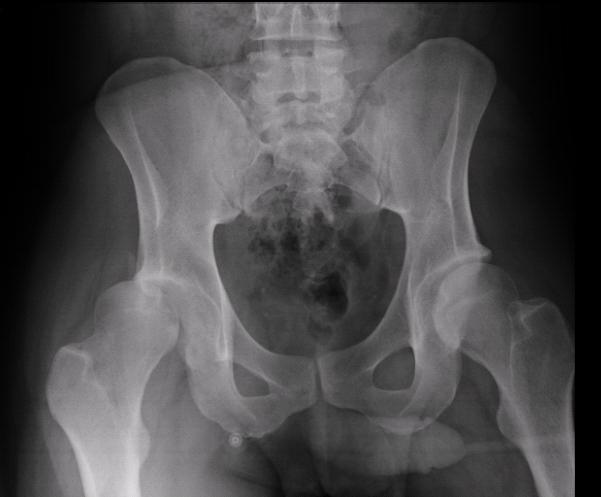**  **Answer:** **Posterior Hip Dislocation** | 10/10 | 9/10 | 10/10 | 7/10 |
| **Case 5b:** How should the emergency management of the above case be?  **a. Immediate reduction is required.**  b. Bed rest, deep vein thrombosis prophylaxis, and a follow-up clinic visit in 1–2 weeks are recommended. | 9/10 | 10/10 | 10/10 | 10/10 |
| **Case 6a:** This elderly patient fell and has right hip pain with shortening and external rotation of the right lower extremity. What is the diagnosis?  **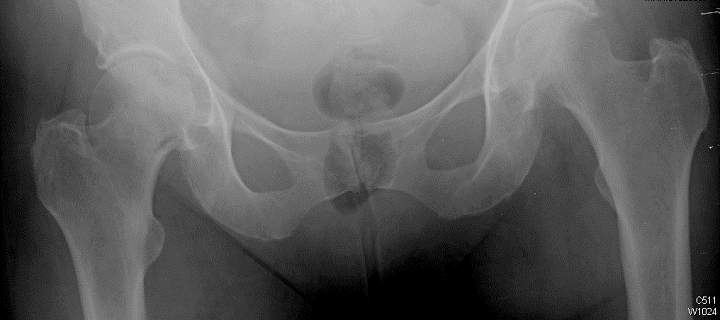Answer: Femoral Neck Fracture** | 10/10 | 9/10 | 10/10 | 8/10 |
| **Case 6b:** How should the emergency management of the above case be?  **a. Emergency surgery should be planned.**  b. Bed rest, deep vein thrombosis prophylaxis, and a follow-up clinic visit in 1–2 weeks are recommended. | 10/10 | 9/10 | 10/10 | 10/10 |
| **Case 7a:** This patient fell backward lifting a box out of a truck and struck his elbow. What is the diagnosis?  **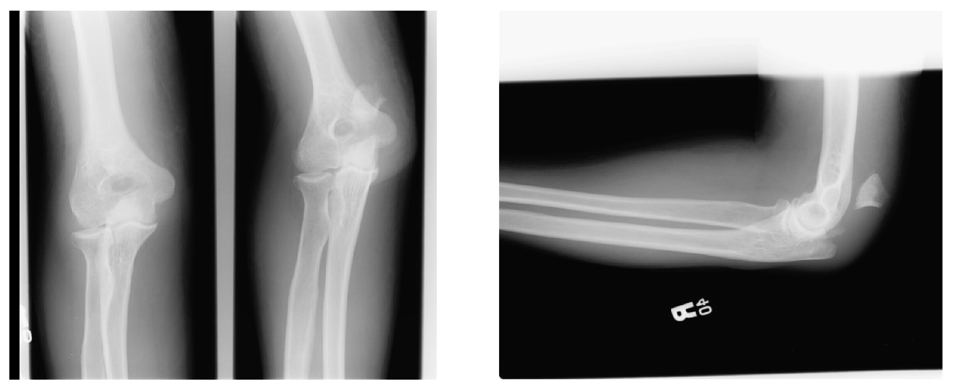**  **Answer: Olecranon Fracture** | 10/10 | 7/10 | 10/10 | 9/10 |
| **Case 7b:** How should the emergency management of the above case be?  **a. Emergency surgery should be planned.**  b. The extremity should be immobilized and the patient should be referred to an orthopedist in 1–2 weeks. | 9/10 | 10/10 | 10/10 | 9/10 |
| **Case 8a:** This patient had an MVC and has severe mid-thigh pain and swelling. What is the diagnosis?  **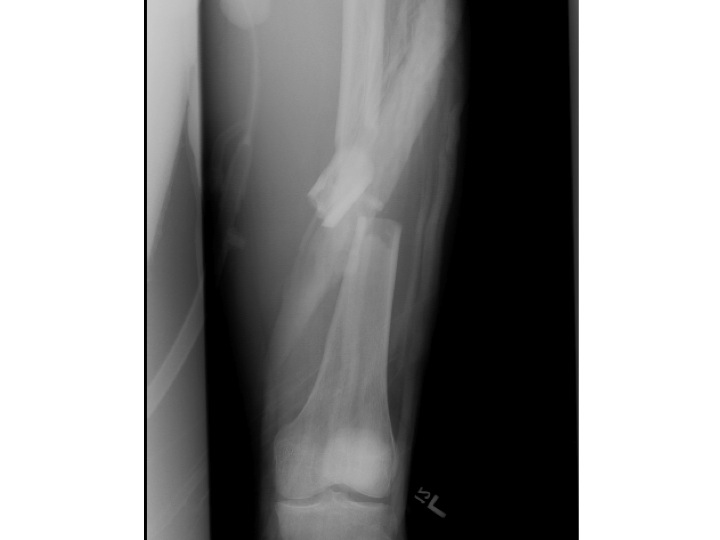**  **Answer:** **Femoral Shaft Fracture** | 10/10 | 9/10 | 9/10 | 10/10 |
| **Case 8b:** How should the emergency management of the above case be?  **a. Emergency surgery should be planned.**  b. The extremity should be immobilized and the patient should be referred to an orthopedist in 1–2 weeks. | 10/10 | 10/10 | 10/10 | 10/10 |
| **Case 9a:** A young male is hit in the lateral leg with a baseball bat during a robbery attempt. He is able to walk, but presents with leg pain. What is the diagnosis?  **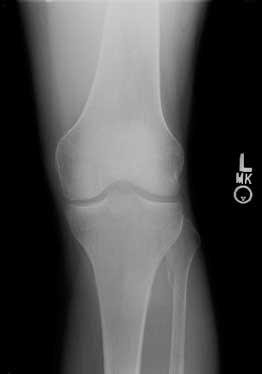**  **Answer:** **Proximal Fibula Fracture** | 10/10 | 9/10 | 9/10 | 0/10 |
| **Case 9b:** How should the emergency management of the above case be?  a. Emergency surgery should be planned.  **b. The extremity should be immobilized and the patient should be referred to an orthopedist in 1**–**2 weeks.** | 9/10 | 6/10 | 9/10 | 10/10 |
| **Case 10a:** An 8-year-old female who fell on her outstretched hand 5 h ago complains of wrist pain. She holds it still but has very little tenderness or swelling on exam. What is the diagnosis?**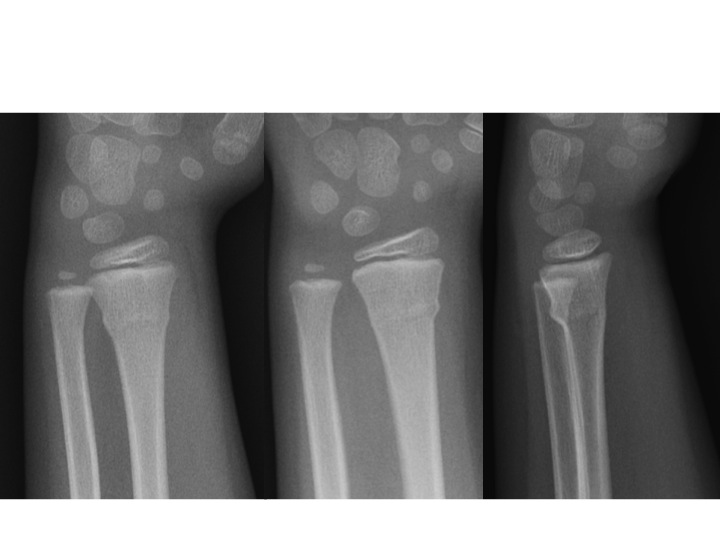**  **Answer: Torus Fracture** | 8/10 | 10/10 | 10/10 | 10/10 |
| **Case 10b:** How should the emergency management of the above case be?  a. Emergency surgery should be planned.  **b. The extremity should be immobilized and the patient should be referred to an orthopedist in 1**–**2 weeks.** | 9/10 | 8/10 | 10/10 | 10/10 |
| EMS: Emergency Medicine Specialist EMR: Emergency Medicine Resident OTS: Orthopedist GPT: GPT-4 omni MVC: Motor Vehicle Collisions | | | | |
